# Rivaroxaban versus enoxaparin/vitamin K antagonist therapy in patients with venous thromboembolism and renal impairment

**DOI:** 10.1186/1477-9560-12-25

**Published:** 2014-11-24

**Authors:** Rupert M Bauersachs, Anthonie WA Lensing, Martin H Prins, Dagmar Kubitza, Ákos F Pap, Hervé Decousus, Jan Beyer-Westendorf, Paolo Prandoni

**Affiliations:** Department of Vascular Medicine, Klinikum Darmstadt GmbH, Grafenstraße 9, 64283 Darmstadt, Germany; Bayer HealthCare, Wuppertal, Germany; University of Maastricht, Maastricht, the Netherlands; Université Jean Monnet, Saint-Etienne, France; Dresden University Hospital “C.G.Carus”, Dresden, Germany; Department of Medicine, University of Padua, Padua, Italy

**Keywords:** Anticoagulants, Bleeding, Renal insufficiency, Rivaroxaban, Venous thromboembolism

## Abstract

**Background:**

Patients with renal impairment receiving classical anticoagulation for venous thromboembolism (VTE) are at increased risk of bleeding and possibly pulmonary embolism. We examined the efficacy and safety of oral rivaroxaban in patients with VTE with and without renal impairment.

**Methods:**

Prespecified subgroup analysis of the EINSTEIN DVT and EINSTEIN PE studies comparing fixed-dose rivaroxaban with enoxaparin/a vitamin K antagonist (VKA), performed in 8246 patients enrolled from 2007 to 2011 in 314 hospitals.

**Results:**

Outcomes were recurrent VTE and major or clinically relevant nonmajor bleeding in patients with normal renal function (n = 5569; 67.3%) or mild (n = 2037; 24.6%), moderate (n = 636; 7.7%), or severe (n = 21; 0.3%) renal impairment. Rates of recurrent VTE were 1.8%, 2.8%, 3.3%, and 4.8% in patients with normal renal function and mild, moderate, and severe renal impairment, respectively (p_trend_ = 0.001). Hazard ratios for recurrent VTE were similar between treatment groups across renal function categories (p_interaction_ = 0.72). Major bleeding in rivaroxaban recipients occurred in 0.8%, 1.4%, 0.9%, and 0%, respectively (p_trend_ = 0.50). Respective rates in enoxaparin/VKA recipients were 1.0%, 3.0%, 3.9%, and 9.1% (p_trend_ < 0.001). Rivaroxaban–enoxaparin/VKA hazard ratios were 0.79 (95% confidence interval [CI] 0.46–1.36) for normal renal function, 0.44 (95% CI 0.24–0.84) for mild renal impairment, and 0.23 (95% CI 0.06–0.81) for moderate renal impairment (p_interaction_ = 0.034).

**Conclusions:**

Patients with symptomatic VTE and renal impairment are at increased risk of recurrent VTE. Renal impairment increased the risk of major bleeding in enoxaparin/VKA-treated patients but not in rivaroxaban-treated patients.

**Trial registration:**

NCT00440193 and NCT00439777.

## Background

Rivaroxaban is an oral, direct Factor Xa inhibitor with predictable pharmacokinetic and pharmacodynamic properties, which obviate the need for routine coagulation monitoring, and a rapid onset of action, with a peak anticoagulant effect within 2 to 4 h after dosing [1, 2]. Pharmacokinetic studies showed that rivaroxaban has a dual mode of elimination; approximately two-thirds of orally administered rivaroxaban is inactivated by metabolic degradation, of which half is eliminated renally and the other half eliminated by the hepatobiliary route. The final one-third of the administered dose undergoes renal excretion as unchanged active substance in the urine, mainly via active renal secretion [3]. Consequently, rivaroxaban exposure increases modestly with declining creatinine clearance (CrCl), with an increase in the area under the plasma concentration–time curve (AUC) of 44%, 52%, and 64% in patients with mild (CrCl 50–79 ml/min), moderate (CrCl 30–49 ml/min), and severe (CrCl <30 ml/min) renal impairment, respectively [4]. The maximum concentrations of rivaroxaban compared with patients with normal CrCl (i.e. ≥80 ml/min) also showed modest increases of 28%, 12%, and 26%, respectively [4]. In addition, the half-life of rivaroxaban was slightly prolonged by 0.4, 0.7, and 1.2 h, respectively [4].

Anticoagulant treatment is associated with an increased risk of bleeding. Because rivaroxaban is partly excreted renally, a dose reduction in patients with renal impairment would seem plausible. However, severe renal impairment has been associated with an increase in the incidence of fatal pulmonary embolism (PE) within the first 2 weeks of diagnosis, which exceeded the risk of fatal bleeding by far, in a prospective registry of patients with symptomatic venous thromboembolism (VTE) treated with low molecular weight heparins and vitamin K antagonists (VKAs) [5]. In addition, moderate or severe renal impairment was recently identified as a risk factor for a first episode of symptomatic venous thrombosis [6]. Consequently, it is important to understand the effect of renal impairment on the efficacy and safety of rivaroxaban in VTE patients to minimize the risk of bleeding while ensuring optimal anticoagulation in patients with renal impairment.

In two large rivaroxaban dose-finding studies in patients with symptomatic deep vein thrombosis (DVT) that evaluated daily rivaroxaban doses between 20 mg and 60 mg, all doses were associated with low rates of recurrent VTE and major bleeding [7, 8]. In addition, there was no increased risk of major bleeding with declining kidney function [8]. As a consequence, the rivaroxaban regimen selected for the phase III studies of VTE treatment consisted of 15 mg twice-daily doses for 3 weeks, followed by 20 mg once daily without dose adaptations for mild-to-moderate renal impairment.

Here, we report on the incidences of recurrent VTE and bleeding in patients with and without renal impairment who participated in the EINSTEIN DVT and EINSTEIN PE studies [9, 10].

## Methods

### Study design

The EINSTEIN DVT and EINSTEIN PE studies were open-label, randomized, event-driven, noninferiority studies that compared oral rivaroxaban alone (15 mg twice daily for 3 weeks, followed by 20 mg once daily) with subcutaneous enoxaparin followed by a VKA (either warfarin or acenocoumarol; target international normalized ratio [INR] 2.0–3.0) for 3, 6, or 12 months in patients with acute, symptomatic DVT and/or PE [9, 10].

Exclusion criteria for both studies were another indication for a VKA; a calculated CrCl <30 ml/min using the Cockcroft–Gault formula [11]; clinically significant liver disease or an alanine aminotransferase level that was three times the upper limit of the normal range or higher; bacterial endocarditis; active bleeding or a high risk of bleeding, contraindicating anticoagulant treatment; systolic blood pressure >180 mm Hg or diastolic blood pressure >110 mm Hg; childbearing potential without proper contraceptive measures, pregnancy, or breastfeeding; concomitant use of strong cytochrome P450 3A4 inhibitors (e.g. human immunodeficiency virus protease inhibitors or systemic ketoconazole) or inducers (e.g. rifampicin, carbamazepine, or phenytoin); participation in another experimental pharmacotherapeutic program within 30 days before screening; and a life expectancy of <3 months.

An independent committee, unaware of treatment assignment, adjudicated all suspected study outcomes. Symptomatic recurrent VTE was defined as a composite of fatal or nonfatal PE or DVT on the basis of criteria that have been described previously [9, 10]. Death was classified as due to PE, bleeding, or other established causes or diagnoses. PE was considered the cause of death if there was objective documentation of the condition or if death could not be attributed to a documented cause and PE could not be confidently ruled out.

Bleeding was classified as major or clinically relevant nonmajor bleeding, as described previously [9, 10, 12]. Bleeding was major if it was clinically overt and was associated with a decrease in the hemoglobin level of ≥2.0 g/dl; if bleeding led to the transfusion of ≥2 units of red cells; or if bleeding was intracranial or retroperitoneal, occurred in another critical site, or contributed to death. Clinically relevant nonmajor bleeding was defined as overt bleeding that did not meet the criteria for major bleeding but was associated with medical intervention, unscheduled contact with a physician, interruption or discontinuation of a study drug, or discomfort or impairment of daily activities.

EINSTEIN study patient data were obtained after informed consent. Ethical approval was obtained from the IRBs of all institutions involved in the EINSTEIN studies [13]. Data from the EINSTEIN studies’ databases had been entirely de-linked from personal health information when accessed for this study. This study is consistent with the principles of the Declaration of Helsinki. Bayer and Janssen sponsored the two EINSTEIN clinical trials, collected and maintained the data, and performed the analyses that the authors requested.

### Statistical analysis

Analyses were performed in SAS version 9.2 (SAS Institute Inc., Cary, NC, USA). Time to efficacy and bleeding outcomes were analyzed using Cox proportional-hazards models stratified according to the intended duration of treatment, with adjustment for absence of active cancer at baseline in each model and including renal function categories, as detailed below. For efficacy, this was based on the intention-to-treat population for the intended treatment period, and for safety, on all patients who received at least one dose of study drug for the period up to 2 days after the last dose.

Renal function was categorized as normal (i.e. CrCl ≥80 ml/min), or with impairment that was mild (CrCl 50–79 ml/min), moderate (CrCl 30–49 ml/min), or severe (CrCl <30 ml/min). The effects of renal function on rates of recurrent VTE and bleeding were tested by including renal function categories in the Cox proportional-hazards models as a single covariate (*χ*^2^ test for trend with 1 degree of freedom). In addition, to test whether this trend was different across treatment groups, the relevant interaction term was included in the model (*χ*^2^ test for interaction of trends by treatment group with 1 degree of freedom). In 9 patients, CrCl values were calculated using the abbreviated Modification of Diet in Renal Disease equation [14], because body weight was not available. Furthermore, separate Cox regression models were fitted to investigate the treatment effect within each renal function category. To allow for comparison between the period of initial bridging treatment with renally cleared low molecular weight heparin alongside hepatically metabolized warfarin or acenocoumarol, and the treatment period with VKA alone, the incidences of outcomes in the population at risk were calculated separately up to Day 14 and for the full study period after Day 14.

## Results

### Patient demographics

In the EINSTEIN DVT and EINSTEIN PE studies combined, 4150 patients were assigned to rivaroxaban and 4131 were assigned to enoxaparin/VKA [13]. A total of 5569 (67.3%) patients had normal renal function (40.5% female; mean age 49.9 years), 2037 (24.6%) patients had mild renal impairment (52.7% female; mean age 69.6 years), 636 (7.7%) patients had moderate renal impairment (65.9% female; mean age 78.2 years), and 21 (0.3%) patients had severe renal impairment (76.2% female; mean age 76.8 years). Values for CrCl were missing in 18 (0.2%) patients and these were not included in the analysis. The main demographic characteristics within the subgroups were similar for rivaroxaban and enoxaparin/VKA (Table 1) [13].

**Table 1:** **Demographic characteristics of EINSTEIN DVT and EINSTEIN PE patients combined**

	Rivaroxaban n = 4150	Enoxaparin/VKA n = 4131
Age, median (Q1–Q3), y	58.0 (45.0–71.0)	59.0 (45.0–70.0)
Female sex, n (%)	1848 (44.5)	1917 (46.4)
Weight, median (Q1–Q3), kg	80.0 (70.0–93.0)	80.0 (70.0–93.0)
Creatinine clearance		
≥80 ml/min, n (%)	2772 (66.8)	2797 (67.7)
Age, median (Q1–Q3), y	50.0 (39.0–61.0)	51.0 (40.0–61.0)
Female sex, n (%)	1113 (40.2)	1140 (40.8)
Weight, median (Q1–Q3), kg	85.0 (74.0–98.0)	85.0 (75.0–98.0)
50–79 ml/min, n (%)	1036 (25.0)	1001 (24.2)
Age, median (Q1–Q3), y	71.0 (64.0–77.0)	71.0 (65.0–77.0)
Female sex, n (%)	520 (50.2)	553 (55.2)
Weight, median (Q1–Q3), kg	74.8 (65.0–82.0)	74.0 (65.0–82.0)
30–49 ml/min, n (%)	323 (7.8)	313 (7.6)
Age, median (Q1–Q3), y	80.0 (75.0–84.0)	79.0 (75.0–83.0)
Female sex, n (%)	209 (64.7)	210 (67.1)
Weight, median (Q1–Q3), kg	67.0 (59.0–75.1)	67.8 (59.0–75.0)
<30 ml/min, n (%)	10 (0.2)	11 (0.3)
Age, median (Q1–Q3), y	80.5 (73.0–86.0)	79.0 (77.0–86.0)
Female sex, n (%)	5 (50.0)	11 (100.0)
Weight, median (Q1–Q3), kg	60.0 (50.0–68.0)	70.0 (48.0–75.0)
Missing, n (%)	9 (0.2)	9 (0.2)
Risk factors for VTE		
Unprovoked VTE, n (%)	2003 (48.3)	2048 (49.6)
Previous VTE, n (%)	791 (19.1)	819 (19.8)
Active cancer, n (%)	232 (5.6)	198 (4.8)

Q, Quartile; VKA, Vitamin K antagonist; VTE, Venous thromboembolism.

### Recurrent venous thromboembolism and renal function

Rivaroxaban was noninferior to enoxaparin/VKA for the prevention of recurrent VTE (rivaroxaban, 86 events [2.1%], enoxaparin/VKA, 95 events [2.3%]; hazard ratio [HR] 0.89; 95% confidence interval [CI] 0.66–1.19; p < 0.001 for noninferiority margin of 1.75) [13, 15].

**Figure 1:**
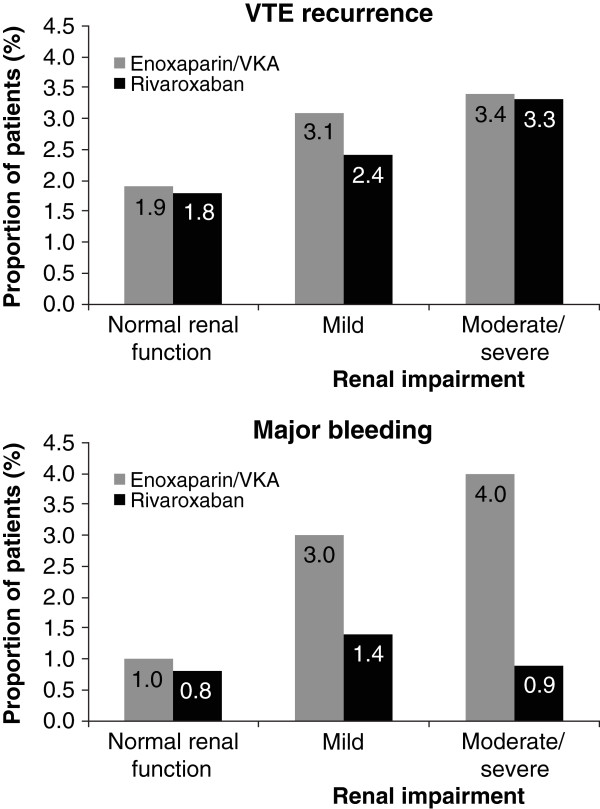
**Recurrent VTE and bleeding in relation to renal function across the entire analysis period.** EINSTEIN DVT and EINSTEIN PE patients. VKA, vitamin K antagonist; VTE, venous thromboembolism.

Rates of recurrent VTE for both treatments combined were 1.8%, 2.8%, 3.3%, and 4.8% in patients with normal renal function and mild, moderate, and severe renal impairment, respectively (p_trend_ = 0.001). The respective incidence rates for rivaroxaban and enoxaparin/VKA patients are shown in Table 2 and Figure 1. The rivaroxaban–enoxaparin/VKA HRs were similar for those with normal renal function (HR 0.95; 95% CI 0.65–1.41), for those with mild renal impairment (HR 0.77; 95% CI 0.45–1.30), and for those with moderate renal impairment (HR 1.05; 95% CI 0.44–2.47), respectively (p_interaction_ = 0.72). In the subgroup of patients with severe renal impairment, no event occurred in the rivaroxaban group and 1 event occurred in the VKA group.

**Table 2:** Recurrent VTE and bleeding in relation to renal function, treatment, and treatment period: EINSTEIN DVT and EINSTEIN PE patients combined*

	Entire analysis period		Period up to 14 days		Period after 14 days	
	Rivaroxaban	Enoxaparin/VKA	Rivaroxaban	Enoxaparin/VKA	Rivaroxaban	Enoxaparin/VKA
	n = 4150	n = 4131	n = 4150	n = 4131	n = 4054	n = 4001
**Recurrent VTE n/N (%)**						
Total of recurrent VTE	86	95	38	37	48	58
Normal renal function	50/2772 (1.8)	52/2797 (1.9)	23/2772 (0.8)	26/2797 (0.9)	27/2720 (1.0)	26/2716 (1.0)
Mild renal impairment	25/1036 (2.4)	31/1001 (3.1)	10/1036 (1.0)	8/1001 (0.8)	15/1009 (1.5)	23/974 (2.4)
Moderate renal impairment	11/323 (3.4)	10/313 (3.2)	5/323 (1.5)	2/313 (0.6)	6/307 (2.0)	8/296 (2.7)
Severe renal impairment	0/10 (0)	1/11 (9.1)	0/10 (0)	1/11 (9.1)	0/10 (0)	0/8 (0)
Missing	0/9 (0)	1/9 (11.1)	0/9 (0)	0/9 (0)	0/8 (0)	1/7 (14.3)
**Major bleeding n/N (%)**						
Total of major bleeding events	40	72	13	27	27	45
Normal renal function	23/2763 (0.8)	29/2786 (1.0)	8/2763 (0.3)	11/2786 (0.4)	15/2689 (0.6)	18/2704 (0.7)
Mild renal impairment	14/1030 (1.4)	30/1002 (3.0)	5/1030 (0.5)	10/1002 (1.0)	9/985 (0.9)	20/954 (2.1)
Moderate renal impairment	3/320 (0.9)	12/310 (3.9)	0/320 (0)	5/310 (1.6)	3/305 (1.0)	7/285 (2.5)
Severe renal impairment	0/9 (0)	1/11 (9.1)	0/9 (0)	1/11 (9.1)	0/9 (0)	0/6 (0)
Missing	0/8 (0)	0/7 (0)	0/8 (0)	0/7 (0)	0/8 (0)	0/7 (0)
**Major and clinically relevant nonmajor bleeding n/N (%)**						
Total of major and clinically relevant nonmajor bleeding events	388	412	129	134	259	278
Normal renal function	239/2763 (8.7)	245/2786 (8.8)	75/2763 (2.7)	73/2786 (2.6)	164/2627 (6.2)	172/2646 (6.5)
Mild renal impairment	110/1030 (10.7)	123/1002 (12.3)	40/1030 (3.9)	40/1002 (4.0)	70/957 (7.3)	83/927 (9.0)
Moderate renal impairment	37/320 (11.6)	43/310 (13.9)	14/320 (4.4)	20/310 (6.5)	23/291 (7.9)	23/272 (8.5)
Severe renal impairment	2/9 (22.2)	1/11 (9.1)	0/9 (0)	1/11 (9.1)	2/9 (22.2)	0/6 (0)
Missing	0/8 (0)	0/7 (0)	0/8 (0)	0/7 (0)	0/8 (0)	0/7 (0)

VKA, Vitamin K antagonist; VTE, Venous thromboembolism.

*Outcomes do not include censored patients or patients who had an event before Day 14.

### Bleeding and renal function

A first major or clinically relevant nonmajor bleeding event occurred in 388 patients (9.4%) in the rivaroxaban group and in 412 patients (10.0%) in the enoxaparin/VKA group (HR 0.93; 95% CI 0.81–1.06; p = 0.27) [13]. In patients receiving rivaroxaban, major and clinically relevant nonmajor bleeding occurred in 8.7% of patients with normal renal function, in 10.7% of those with mild renal impairment, in 11.6% of those with moderate renal impairment, and in 22.2% of those with severe renal impairment (p_trend_ = 0.013). In the enoxaparin/VKA group, these incidences were 8.8%, 12.3%, 13.9%, and 9.1%, respectively (p_trend_ < 0.001) (Table 2). The rivaroxaban–enoxaparin/VKA HR was 0.98 (95% CI 0.82–1.18) for patients with normal renal function, 0.85 (95% CI 0.65–1.09) for those with mild renal impairment, and 0.77 (95% CI 0.49–1.19) for those with moderate renal impairment (p_interaction_ = 0.29).

Major bleeding occurred in 40 (1.0%) rivaroxaban and 72 (1.7%) enoxaparin/VKA recipients (HR 0.54; 95% CI 0.37–0.79; p = 0.002; Table 3) [13]. In patients receiving rivaroxaban, major bleeding occurred in 0.8% of patients with normal renal function, in 1.4% of those with mild renal impairment, in 0.9% of those with moderate renal impairment, and in 0% of those with severe renal impairment (p_trend_ = 0.50). In the enoxaparin/VKA group, these incidences were 1.0%, 3.0%, 3.9%, and 9.1%, respectively (p_trend_ < 0.001) (Table 2). The rivaroxaban–enoxaparin/VKA HRs were 0.79 (95% CI 0.46–1.36) for those with normal renal function, 0.44 (95% CI 0.24–0.84) for those with mild renal impairment, and 0.23 (95% CI 0.06–0.81) for those with moderate renal impairment (p_interaction_ = 0.034).

**Table 3:** **Presentation of major bleeding for rivaroxaban and enoxaparin/VKA patients separately**

	Rivaroxaban	Enoxaparin/VKA
	n = 4130	n = 4116
First major bleeding, n (%)		
Any	40 (1.0)	72 (1.7)
Fatal bleeding	3 (<0.1)	8 (0.2)
Retroperitoneal	0	1 (<0.1)
Intracranial	2 (<0.1)	4 (0.1)
Gastrointestinal	1 (<0.1)	2 (<0.1)
Thorax	0	1 (<0.1)
Nonfatal bleeding in a critical site	10 (0.2)	27 (0.7)
Retroperitoneal	1 (<0.1)	7 (0.2)
Intracranial	3 (<0.1)	9 (0.2)
Intraocular	3 (<0.1)	3 (<0.1)
Pericardial	0	2 (<0.1)
Intra-articular	0	4 (0.1)
Adrenal	1 (<0.1)	0
Pulmonary	1 (<0.1)	0
Abdominal	1 (<0.1)	2 (<0.1)
Nonfatal, noncritical site bleeding but associated with a fall in hemoglobin ≥ 2 g/dl and/or transfusions ≥2 units	27 (0.7)	37 (0.9)
Surgical site	0	3 (<0.1)
Skin	1 (<0.1)	5 (0.1)
Urogenital	9 (0.2)	3 (<0.1)*
Gastrointestinal	14 (0.3)	24 (0.6)
Nasal	1 (<0.1)	0
Pulmonary	1 (<0.1)	0
Intramuscular	1 (<0.1)	2 (<0.1)

VKA, Vitamin K antagonist.

*One patient had a combined gastrointestinal/urogenital bleeding event; this event is counted as gastrointestinal only.

The risk of major bleeding was significantly increased in renally impaired patients treated with enoxaparin/VKA, whereas renal impairment across all stages did not increase major bleeding rates in patients treated with rivaroxaban. The reduction of major bleeding seen in the rivaroxaban group was similarly achieved during the initial treatment period, compared with enoxaparin/VKA treatment (treatment period up to 14 days), and during long-term treatment, compared with VKA alone (treatment period after 14 days; Table 2).

## Discussion

This analysis of the data accumulated in the EINSTEIN DVT and EINSTEIN PE studies indicated that the risks of recurrent VTE and bleeding increase with declining renal function. In addition, the results demonstrated that a dosage of rivaroxaban 15 mg twice daily for 3 weeks, followed by 20 mg once daily, had similar efficacy compared with standard treatment across patients with normal renal function or mild-to-moderate renal impairment. Incidences of the combined outcome of major or clinically relevant nonmajor bleeding were numerically lower with rivaroxaban compared with enoxaparin/VKA. Even more important, there was a significant and clinically important reduction in major bleeding with rivaroxaban compared with enoxaparin/VKA, particularly in patients with mild or moderate renal impairment.

In the EINSTEIN Extension study that compared 20 mg rivaroxaban once daily with placebo for an additional 6 or 12 months in patients who had completed 6–12 months of treatment for VTE, an increased risk of recurrent VTE with declining renal function was also shown [9]. In rivaroxaban recipients with normal renal function or mild, moderate, or severe renal impairment, recurrent VTE occurred in 1.0% (4/409), 1.4% (2/147), 4.9% (2/41), and 0%, respectively (p_trend_ = 0.045). In placebo recipients, recurrent VTE occurred in 6.2% (25/404), 8.0% (11/138), 10.9% (5/46), and 20.0% (1/5), respectively (p_trend_ = 0.045) [9]. On the other hand, rates of major or clinically relevant nonmajor bleeding in patients receiving rivaroxaban did not significantly increase with declining renal function, since bleeding occurred in 6.4% (26/406), 6.2% (9/146), 2.4% (1/41), and 0% of rivaroxaban recipients with normal renal function or mild, moderate, or severe renal impairment, respectively (p_trend_ = 0.282). In the placebo group, these incidences were 0.7% (3/402), 0.7% (1/137), 4.3% (2/46), and 20.0% (1/5), respectively (p_trend_ = 0.031) [9].

Because approximately one-third of the administered dose of rivaroxaban is eliminated via the kidneys (as unchanged drug) [3], whereas enoxaparin is cleared almost exclusively by the kidneys [16–18], and warfarin/acenocoumarol by the liver [19–21], it could be expected that the reduction in major bleeding associated with rivaroxaban would be largely obtained during the period of initial treatment during which patients typically receive enoxaparin. However, the distribution of major bleeding over time suggests that the reduced incidence of major bleeding with rivaroxaban is present both during the initial treatment period, when compared with enoxaparin/VKA treatment, and during long-term treatment, when compared with VKA alone (Table 2). The potential mechanisms behind this observation are subject to speculation. Foremost, in patients with severe renal impairment, rivaroxaban exposure (expressed as AUC) increases by ~60%, whereas the maximum plasma concentration increases by only ~30%, well within its therapeutic window [4]. In addition, the half-life of rivaroxaban increases by only ~1 h in patients with severe renal impairment, indicating a limited potential for drug accumulation [4]. These modest increases might be the result of a higher fraction of rivaroxaban cleared via several independent hepatic pathways. In addition, patients with renal impairment have been reported to have a higher sensitivity to VKAs, thus requiring smaller dosages and more intensive INR monitoring [21–23]. However, in the EINSTEIN DVT and EINSTEIN PE studies, the time spent above an INR of 3.0 was 15.2% for patients with normal renal function, 17.6% for patients with mild renal impairment, and 17.9% for patients with moderate renal impairment; therefore, a higher sensitivity to VKAs in renally impaired patients cannot fully explain the observed increase in incidence of major bleeding with declining renal function, and further research needs to be done to address this issue.

Some methodological aspects of our analysis warrant comment. First, the analyses of efficacy and safety in subgroups according to renal function were specified *a priori*. Data were collected prospectively with central adjudication of clinical events by assessors unaware of treatment assignment. The number of patients with mild renal impairment was large, totaling over 2000 patients, whereas the number with moderate renal impairment was substantially smaller, at approximately 600 patients. Severe renal impairment, defined as CrCl <30 ml/min, was an exclusion criterion, and few of these patients were randomized. The data collection did not account for significant changes in patients’ renal function over the course of treatment.

We identified an increased risk of bleeding in renally impaired patients receiving anticoagulation, but a limitation of our study was the exclusion from participation in the studies of patients with a high bleeding risk or severe renal impairment. Therefore, bleeding rates may be even higher in unselected patients. Owing to the very limited number of patients with severe renal impairment, more cases need to be studied to determine the rate of bleeding.

What are the clinical implications of the present findings? In patients presenting with symptomatic VTE and mild-to-moderate renal impairment, large phase III trials have provided support that rivaroxaban can be administered at a fixed dose without adjustment for renal function and carries a safety advantage compared with standard treatment with enoxaparin/VKA, while maintaining efficacy. The limited increases in exposure, maximum concentration, and half-life of rivaroxaban with declining renal function are similar to those of apixaban [24], but are in contrast to dabigatran, for which exposure increases by 50%, 215%, and 530% and half-life by 2.8, 4.9, and 13.7 h in patients with mild, moderate, and severe renal impairment, respectively [25].

In patients who have a long-term indication for anticoagulation, the use of rivaroxaban seems to be attractive because it may offer a broad safety window for patients with declining renal function that is not covered by the regular monitoring of renal function, which is usually recommended. For patients with severe renal impairment, little evidence is available. Rivaroxaban is not recommended in patients with CrCl <15 ml/min, and it should be used with caution in patients with CrCl 15–29 ml/min.

## Conclusions

We conclude that both recurrent venous thromboembolic complications and the risk of bleeding increase with declining kidney function in patients with symptomatic DVT or PE. The standard regimen of rivaroxaban, given without a dose reduction, is efficacious and associated with a lower incidence of major bleeding compared with treatment with enoxaparin/VKA.

## Abbreviations

AUC: Area under the concentration–time curve
CI: Confidence interval
CrCl: Creatinine clearance
DVT: Deep vein thrombosis
HR: Hazard ratio
PE: Pulmonary embolism
Q: Quartile
VKA: Vitamin K antagonist
VTE: Venous thromboembolism.
